# First-time seizure revealing late-onset Fahr’s disease: a case report and brief literature review

**DOI:** 10.3389/fnhum.2024.1456610

**Published:** 2024-11-22

**Authors:** Adugna Lamessa, Kenna Tesfaye, Tamirat Godebo Woyimo, Ermias Habte Gebremichael

**Affiliations:** Department of Internal Medicine, Jimma University, Jimma, Ethiopia

**Keywords:** Fahr’s disease, rare, seizure, intracerebral calcification, Ethiopia

## Abstract

Fahr’s disease (FD), otherwise known as primary familial brain calcification, is a rare neurodegenerative condition that involves intracerebral calcification at the level of the basal ganglia and other brain regions. It is an inherited neurologic disorder, although its molecular genetics have not been thoroughly defined. Patients usually present with a wide range of symptoms, predominantly movement disorders and cognitive changes. However, seizures are a rare initial presenting features of late-onset FD in adults. Herein, we present the case of a 60-year-old man with no known chronic illnesses who was admitted to a tertiary hospital after experiencing first-time generalized tonic-clonic seizures and loss of consciousness for two days. Basic laboratory results were within normal limits, and a non-contrast brain computed tomography (CT) scan showed intracerebral calcification. The patient was diagnosed with epilepsy secondary to FD based on its modified diagnostic criteria and responded well to antiepileptic treatment. The case highlights a rare association and emphasizes the importance of considering this diagnosis in patients experiencing an inaugural seizure; appropriate tests should be performed to confirm or rule out other relevant and secondary causes, and the treatment should be modified accordingly.

## Introduction

Fahr’s disease (FD), otherwise known as primary familial brain calcification, is an uncommon neurological disorder, either inherited or sporadic, characterized by bilateral and symmetrical progressive calcifications in the different parts of brain regions, particularly the basal ganglia and subcortical white matter. These result from abnormal calcium deposits caused by disruptions of calcium-phosphate metabolism and perturbations of the blood-brain barrier integrity (Abubakar and Saidu, 2012; Adekanmi et al., 2020; Tai and Batla, 2015). Epidemiologically, it is a rare disorder that affects fewer than one in a million (Saleem et al., 2013). However, available data indicated that the current estimated prevalence rate ranges from 2.1 to 6.6 per 1,000 individuals (Chen et al., 2023). FD was first described by Karl Theodor Fahr in 1930 (Saleem et al., 2013; Donzuso et al., 2019; Chen, 2022). It primarily affects individuals in the third to fifth decades of their lives but can also occur later in adulthood (Saleem et al., 2013; Nicolas et al., 2015). Majority of the cases present with movement disorder or extrapyramidal symptoms. However, neurological features such as seizures as an initial presentation of late-onset FD are quite unusual (Ayoub et al., 2020; Sudan et al., 2022; Aldawsari et al., 2021).

In Ethiopia, no FD cases have been documented previously, making this the first to be reported in an adult patient. Moreover, there is limited data on FD from Africa, with only a handful of cases have been published (Abubakar and Saidu, 2012; Adekanmi et al., 2020; Ayoub et al., 2020). In the case presented here, we highlight the importance of considering FD in the differential diagnosis of elderly patients presented with inaugural seizure and unexplained intracerebral calcifications on the brain imaging. Furthermore, the case sheds light on its clinical implications for healthcare providers, being the first case documented in the country.

### Case presentation

Herein, we are reporting the case of a 60-year-old male patient with no known chronic illnesses who presented to teaching hospital with generalized tonic–clonic seizures and loss of consciousness for two days. The abnormal movements occurred on more than four occasions, each lasting approximately 10 to 15 minutes, accompanied by upward eye deviation and drooling. The patient also reported no regaining of consciousness between seizure episodes, along with experiencing urinary incontinence, headache and vomiting.

Otherwise, there was no history of fever, neck stiffness, cough, dysarthria, dementia, muscle spasticity, tremors, or body weakness. Additionally, there were no reports of skin color changes such as alopecia, photosensitivity, or oral lesions. He denied any previous seizures or family history of similar episodes. Drugs like chronic use of proton pumping inhibitors, immunosuppressive therapies, and trauma histories were unremarkable. A thorough psychiatric assessment showed no evidence of anxiety, psychosocial stress, or depression.

A medical evaluation revealed a stable vital sign. BP: 110/75 mmHg, PR: 90 bpm, RR: 24 breaths/min, Temperature: 37.5°C, oxygen saturation: 94% on room air. The general examination was normal except for the neurological examination, which revealed a Glasgow Coma Scale (GCS) of 13 out of 15 (E-3, V-4, M-6) upon admission. The pupils were normal and midsize in both eyes. Strength was normal, with a normotonic in the limbs. Deep tendon reflexes were 2 out of 4 in the patellar and ankle bilaterally, with down-going plantar reflexes. Cranial nerves were intact. In addition to the motor and reflex examinations, the other neurological examination findings, including sensory function and higher mental functions were normal. On examination of the cerebellar functions, there was a normal gait with no tremor, dysdiadochokinesis, or ataxia.

### Investigations and treatment of the case

To further refine the differential diagnosis, laboratory tests were performed. A complete blood count (CBC), organ function tests, random blood glucose, thyroid function test (TSH), and coagulation profile were all within the normal range. The serum electrolytes showed ionized calcium at 1.25 (1.20–1.40 mmol/L) and phosphorus at 4 mg/dL. Vitamin D level was 29.55 (30–100 ng/mL) and parathyroid hormone was 29 (15–70 pg./mL), all within normal ranges. The Venereal Disease Research Laboratory (VDRL) and human immunodeficiency virus (HIV) tests were nonreactive (Table 1).

**Table 1:** Laboratory data on admission, 2024.

Laboratory variables	Laboratory data		References range
	25/1/2024	30/1/2024	
White cell count (per μL)	14,800	6780	4,000-15,000
Differential cell count			
Neutrophil (%)	91	69.6	37–72
Lymphocyte (%)	4	18.4	20–50
Hemoglobin (mg/dL)	10.7	10.1	11–16.5
Hematocrit (%)	28.7	37.9	33–49.5%
Platelet count (per μL)	763,000	394,000	150,000–450,000
Creatinine (mg/dL)	1.0	0.93	0.5–1.2
Sodium (mmol/L)	137		135–145
Potassium (mmol/L)	4.5		3.5–5.5
Chloride (mmol/L)	100		98–107
Total calcium (mmol/L)	2.25	2.35	2.2–2.7
Ionized calcium (mmol/L)	1.25		1.20–1.40
25-(OH) vitamin D (ng/mL)	29.55		30–100
Phosphorus (mg/dL)	4		2.7–4.5
Magnesium level (mmol/L)	0.7		0.65–1.05
Parathyroid hormone (pg/mL)	29		15–70
AST (IU/L)	85		0–40
ALT (IU/L)	35		0–41
ALP (IU/L)	73		40–130
Random blood glucose (mg/dL)	170	120	
Prothrombin time (sec)	12.5		10–15
Activated partial thromboplastin time (sec)	28		22–38
International normalized ratio	1.01		0.7–1.3
TSH (μIU/mL)	2.2		0.27–4.2
CSF analysis	Unremarkable		
HIV test	Non-reactive		
VDRL test	Non-reactive		
Hepatitis C antibody test and hepatitis B surface antigen	Negative		
Urinalysis	Unremarkable		

AST/ALT, aspartate transaminase/alanine transaminase; ALP, alkaline phosphatase; CSF, Cerebrospinal Fluid; VDRL, Venereal Disease Research Laboratory; TSH, thyroid stimulating hormone; HIV, human immunodeficiency virus.

**Figure 1:**
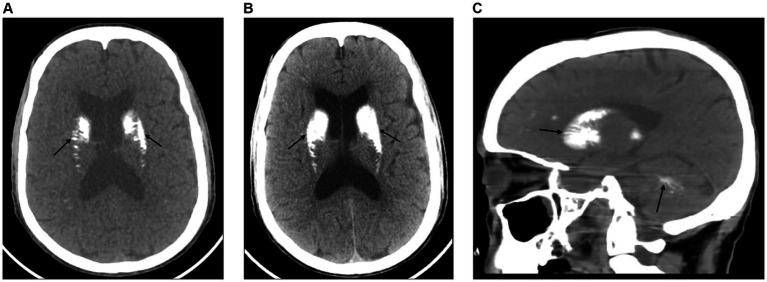
**(A)** An axial view of the brain CT scan shows bilateral symmetric calcifications of the basal ganglia, thalamus, dentate nucleus, and subcortical white matter with no midline shift (black arrows). **(B)** An axial view of the brain CT scan shows bilateral symmetric dense calcifications of the basal ganglia, thalamus, dentate nucleus, and subcortical white matter. There is also prominence of the lateral ventricles with no midline shift (black arrows). **(C)** A sagittal view of the brain CT scan shows dense calcifications within the basal ganglia, corona radiata, and deep white matter of the cerebellum (black arrows).

The brain CT scan revealed bilateral symmetric intracerebral dense calcifications. No hemorrhagic or ischemic lesions were detected (Figures 1A–C). The chest X-ray and abdominal ultrasound were also unremarkable. Thereafter, based on the proposed diagnostic criteria, a diagnosis of epilepsy secondary to FD was established. Given the characteristic clinical features, biochemical analysis, and imaging findings noted earlier, there were no other specific causes identified. The patient was then immediately admitted to the stroke unit and initiated on diazepam 10 mg intravenous infusion, oral phenytoin 100 mg thrice a day and supportive care. Under comprehensive medical supervision, the seizures were efficiently controlled and exhibited a favorable response to the treatment.

### Outcome and follow-up

During the subsequent hospital stay, the patient showed marked improvement in mental status and responded well to antiepileptic therapy. He was discharged in stable condition after one month of admission, prescribed phenytoin 100 mg three times a day and no seizures reported during follow-up. The patient complied with the medication regimen and experienced no adverse effects. Two weeks later (while in the hospital), the patient underwent a reimaged brain CT scan to rule out the possibility of a missed ischemic stroke; however, the finding was similar. Furthermore, to better understand the type and causes of the seizures, the physicians offered patient-centered discussion on the benefit of further workup, but the patient declined additional brain imaging such as an electroencephalogram (EEG) and magnetic resonance angiography (MRA), opted to continue with his medications. This presents a limitation of the case.

## Discussion

Fahr’s disease (FD) is a condition involving non-atherosclerotic intracerebral calcification in the basal ganglia and other brain regions. This rare genetic neurodegenerative disorder is primarily inherited as an autosomal dominant trait, although sporadic or autosomal recessive occurrences are also possible (Donzuso et al., 2019; Peters et al., 2020).

The exact causes of FD are not fully elucidated, with limited evidence at the molecular and genetic levels. While most FD cases are associated with genetic mutations, the cause remains unidentified in approximately one-third of instances. Key initial genes involved in FD include mutations in SLC20A2 [sodium-dependent phosphate transporter 2 (PiT-2)] on chromosome 8 and PDGFRB (Platelet Derived Growth Factor Receptor Beta) genes, which are important for upholding the integrity of the blood–brain barrier (Saleem et al., 2013; Keasey et al., 2016; Amisha and Munakomi, 2023). Most studies indicate that mutations in the SLC20A2 gene (40%) cause dysregulation of calcium and phosphate metabolism, which often plays a role in the pathogenesis of FD. Additionally, the mutations in the genes PDGFB (Platelet Derived Growth Factor Beta) and XPR1 (xenotropic and polytropic retrovirus receptor 1) are also implicated (Saleem et al., 2013; Nicolas et al., 2015; Peters et al., 2020).

FD is a slowly progressive condition that ranges from benign to potentially life-threatening. Patients typically exhibit extrapyramidal symptoms like movement disorders as well as neuropsychiatric features such as memory loss, personality changes, delusions, hallucinations, and depression. Movement disorders, notably parkinsonism, ataxia, and cognitive decline, are the most common presentations (Tai and Batla, 2015; Donzuso et al., 2019; Perugula and Lippmann, 2016).

FD has two distinct clinical forms, as documented by few studies: early-onset (under 40 years) and late-onset (over 50 years) (Iqbal et al., 2022; Aghemo et al., 2023). In late-onset FD, the movement disorder preceding dementia-like symptoms was noted to manifest earlier, probably due to progressive neurodegeneration (Iqbal et al., 2022). In contrast, the early-onset form usually presents with the opposite symptoms sequence (Aghemo et al., 2023). However, the neurological symptoms of FD are extremely uncommon as an initial presentation in an elderly patient (Tai and Batla, 2015; Ayoub et al., 2020; Okike et al., 2021; Thillaigovindan et al., 2019). In a literature review, seizures are considered rare presenting features that aid in the diagnosis of FD and may not be present in some patients (Sudan et al., 2022; Thillaigovindan et al., 2019; Amin, 2013; Hastalığı, 2013). The mechanism causing these symptoms is not clearly explained. Some evidence suggests that the possible dysfunction of cortico-basal connections and their interhemispheric relation plays a key role in the development of FD clinical features as documented by Choayb et al. (2023). In addition, seizure was thought to be due to neuronal damage caused by calcification as reported by Okike et al. (2021) and Thillaigovindan et al. (2019) (Table 2).

**Table 2:** Summary of relevant literature review shows link between seizures and FD over the past decade (2013–2023), 2024.

References	Age/Sex/case	Year	Imaging finding	Associated relevant causes	Seizure
Choayb et al., 2023	47/M/1	2023	Intracerebral calcification (BG, SC, DN, Th)	–	Yes
Hastalığı, 2013	34/M/1	2013	Intracerebral calcification (BG, SC, DN, Th)	–	Yes
Amin, 2013	34/M/1	2013	Intracerebral calcification (BG, SC, Th)		Yes
Okike et al., 2021	9/F/1	2021	Intracerebral calcification (BG, SC, DN, Th)	–	Yes
Otu et al., 2015	58/M/1	2015	Intracerebral calcification (BG, SC, Th)		Yes
Aldawsari et al., 2021	35/F/1	2021	BGC	–	Yes
Ayoub et al., 2020	45/M/1	2020	BGC	–	Yes
Mujuru et al., 2016	11/F/1	2017	BGC	–	Yes

BGC, Basal Ganglia calcification; DN, Dentate Nucleus; SC, Subcortical white matter; Th, Thalami; M, Male; F, Female; 1, Number of cases.

Delays in the diagnosis of FD are frequent. Diagnosis requires a distinctive imaging finding, characteristic clinical features, and the exclusion of known underlying secondary causes (Perugula and Lippmann, 2016). Evidence suggests that brain CT scan is the preferred neuroimaging for the diagnosis of FD, revealing bilateral, symmetrical intracerebral calcifications in the basal ganglia and cerebral cortex (Rastogi et al., 2011; Gligorievski, 2018). Molecular genetic testing of FD is required for the diagnosis, which is limited to an index case where genetic confirmation is needed after excluding secondary etiologies of basal ganglia calcification (Saleem et al., 2013; Amisha and Munakomi, 2023; Moskowitz et al., 1971). The diagnostic criteria for FD have been proposed by Moskowitz et al. (1971); Ellie et al. (1989), and Manyam (2005) and modified by Saleem et al. (2013) and Perugula and Lippmann (2016). This modified diagnostic criteria include bilateral calcification of the basal ganglia (present on imaging), neurological dysfunction (seizures in this case), age at onset, and absence of biochemical abnormalities (normal calcium and phosphate levels). Our patient met 5 of the 6 criteria (excluding family history), confirming the diagnosis of FD. These criteria are practical to use when the limited availability of advanced diagnostic tools such as genetic testing (Table 3).

**Table 3:** Modified diagnostic criteria of FD.

1.	Bilateral calcification of the basal ganglia and other brain regions visualized on neuroimaging.
2.	Progressive neurologic dysfunction, which generally includes a movement disorder and/or neuropsychiatric manifestations.
3.	Age of onset is typically in the fourth or fifth decade, although this may also present in childhood.
4.	Absence of biochemical abnormalities and somatic features suggestive of a mitochondrial or metabolic disease or other systemic disorder
5.	Absence of an infectious, toxic, or traumatic cause.
6.	Family history consistent with autosomal dominant inheritance.

Source: Adapted with permission from Saleem et al. (2013). Springer Nature, 2024.

### Differential diagnosis

The differential diagnosis of FD is crucial in clinical evaluation, given the overlapping cause with various etiologies of intracerebral calcification. FD shares clinical features such as movement disorder-like features, neuropsychiatric symptoms, and other central nervous system features with endocrine disorders, trauma, toxins, brain infections, and normal aging, requiring a comprehensive approach to differentiation. An important factor is the underlying secondary causes, since FD has no identified primary etiologies. Differentiating FD from other causes of intracerebral calcification, especially endocrine disorders and infectious diseases, is crucial.

More importantly, FD should be distinguished from Fahr’s syndrome. In a literature review, it is usually used as a synonym for FD, but it has been termed Fahr’s syndrome (secondary forms) with a known underlying secondary cause where the main etiologies are an endocrine disorder, mitochondrial myopathy, systemic disease, brain infections, toxic, or traumatic causes (Tai and Batla, 2015; Chen, 2022).

In fact, the most common metabolic disorder closely associated with intracerebral calcification is endocrine disorders. These include idiopathic hypoparathyroidism, secondary hypoparathyroidism (post-thyroidectomy) and pseudohypoparathyroidism. A literature review found that idiopathic and secondary hypoparathyroidism are linked to intracerebral calcification in 23.3% and 15.3% of cases, respectively (Saleem et al., 2013; Amisha and Munakomi, 2023; Berrabeh et al., 2023). Interestingly, biochemical analysis of serum parathyroid hormone, phosphorus, and calcium levels helps to rule out parathyroid abnormalities. In the case presented here, the reassuring results indicated this diagnosis less likely.

Brain infections are recognized as potential causes of intracerebral calcification due to chronic inflammation or granulomatous changes. Notable neuroinfectious diseases include toxoplasmosis, acquired immune deficiency syndrome (AIDS), brucellosis (which may cause asymmetrical calcification), and neurocysticercosis, along with congenital infections from the TORCH complex (toxoplasmosis, rubella, cytomegalovirus, and herpes simplex virus) (Amisha and Munakomi, 2023). However, the first two were less likely diagnoses, as evidenced by the pattern of calcifications (non-ring-enhancing) and seronegative for the HIV test respectively. Additionally, one study indicated debatable, low-quality evidence linking acquired infections to basal ganglia calcification in adults (Snijders et al., 2024).

Toxin exposures, particularly high levels of vitamin D, lead, mercury, ionizing radiation, carbon monoxide, and methotrexate, have been documented as causes of FD-like intracranial calcifications. Prolonged exposure to these substances can lead to neurotoxicity (neural necrosis) (Sanders et al., 2009), resulting in metabolic disruptions and calcifications primarily in the basal ganglia. A few case reports suggested that prolonged lead exposure may contribute to unexplained intracranial calcification in adults (Reyes et al., 1986; Chokshi et al., 2014).

Similarly, Neurolupus, a form of systemic lupus erythematosus affecting the nervous system, has been reported to be linked with brain calcification in 30% of patients, typically identified as diffuse calcification on brain imaging. The most common areas of calcification are the basal ganglia, centrum semiovale, cerebellum, and cerebral cortex (Zhao et al., 2023; Nordstrom et al., 1985).

Furthermore, a review article found that repeated head injuries, especially in children, is associated with a higher incidence of basal ganglia calcification (45.9%), leading to dystrophic calcification in the brain. Over time, post-traumatic calcifications may mimic those seen in FD on imaging studies; however, a thorough evaluation of the patient’s trauma history and calcification patterns can help clinicians distinguish between the two. Older children and adults are less likely to develop intracranial calcification after mild brain trauma (Jiang et al., 2020).

Idiopathic seizures are also important differential diagnosis in elderly patients, accounting for one-third to one-half of cases. This challenges the diagnosis of seizures related to FD; nevertheless, the presence of brain imaging evidence for calcification made less likelihood of this diagnosis (Savino et al., 2016; Liu et al., 2016; Table 4).

**Table 4:** Etiological classification of basal ganglia calcification (BGC).

Classification	Possible etiologies	Disease condition
Primary forms of BGC	Genetic forms including autosomal dominant, familial and sporadic forms.	Fahr’s disease (primary familial brain calcification)
Secondary forms of BGC	Metabolic disorder (hypoparathyroidism, pseudohypoparathyroidism) Infections (brucellosis, AIDS, Toxoplasmosis, TORCH complex) Toxic exposure (Lead, carbon monoxide, mercury and methotrexate) Systemic diseases (Neurolupus) Mitochondrial disease Coat’s syndrome (eye disorder) Neurodegenerative conditions (neuroferritinopathies) Plus Unique FD calcification patterns observed on brain imaging studies.	Fahr’s syndrome

### Treatment

At present, FD is not a remediable condition. Evidence-based treatment strategies are lacking that reverse the course of calcification. The foundational to the treatment of FD is to control the symptoms, prevent complications, and enhance physical rehabilitation (Amisha and Munakomi, 2023; di Biase and Munhoz, 2016). Several studies explored that the seizures usually respond well to antiepileptic drugs (as in our case) (Lauterbach, 2008).

The long-term outcome of FD is unpredictable and variable in between studies. Notably, to date, there’s no definitive cure. Nevertheless, evidence suggests that treating symptoms with antiepileptic, antiparkinsonian, and antipsychotic drugs, tailored to the clinical presentation, can enhance patient outcome and quality of life (Tai and Batla, 2015; Amisha and Munakomi, 2023; Savino et al., 2016).

## Strength and limitation

Our case report highlights several strengths, including detection, raising awareness of a rare case, and a careful evaluation that helped differentiate it from other potential causes. However, it does have some limitations, such as the absence of follow-up electroencephalograms (EEG), magnetic resonance angiography (MRA), and genetic analysis.

## Conclusion

FD is a rare neurological disorder marked by basal ganglia calcification, commonly presenting with movement disorders and cognitive changes. Nonetheless, it is quite uncommon to present with an inaugural seizure as the main symptom of late-onset FD. Moreover, imaging permits an accurate diagnosis in accordance with its proposed diagnostic criteria. Regardless of its rarity, FD should be considered in the differential diagnosis of adult patients with first-time seizures and unexplained basal ganglia calcification when other secondary causes, such as endocrine disorders, infections, and toxins, are ruled out. Given the limited availability of advanced diagnostic tools such as genetic testing in Ethiopia, this case underscores the importance of relying on clinical and radiological criteria. It also highlights the need for greater awareness of rare neurological conditions among healthcare providers.

## Data Availability

The datasets presented in this article are not readily available because of ethical and privacy restrictions. Requests to access the datasets should be directed to the corresponding author.
